# Possible regulation of Toll-like receptor 4 by lysine acetylation through LPCAT2 activity in RAW264.7 cells

**DOI:** 10.1042/BSR20220251

**Published:** 2022-07-15

**Authors:** Victory Ibigo Poloamina, Wondwossen Abate, Gyorgy Fejer, Simon K. Jackson

**Affiliations:** 1 University of Plymouth, Faculty of Health, School of Biomedical Sciences, Plymouth PL4 8AA, U.K.; 2 MolEndoTech Ltd, Brixham TQ5 8BA, U.K.

**Keywords:** acetylation/deacetylation, acetyltransferases, bioinformatics, immunomodulation, inflammation, toll-like receptors

## Abstract

Inflammation is central to several diseases. TLR4 mediates inflammation by recognising and binding to bacterial lipopolysaccharides and interacting with other proteins in the TLR4 signalling pathway. Although there is extensive research on TLR4-mediated inflammation, there are gaps in understanding its mechanisms. Recently, TLR4 co-localised with LPCAT2, a lysophospholipid acetyltransferase. LPCAT2 is already known to influence lipopolysaccharide-induced inflammation; however, the mechanism of LPCAT2 influencing lipopolysaccharide-mediated inflammation is not understood.

The present study combined computational analysis with biochemical analysis to investigate the influence of LPCAT2 on lysine acetylation in LPS-treated RAW264.7 cells.

The results suggest for the first time that LPCAT2 influences lysine acetylation in LPS-treated RAW264.7 cells. Moreover, we detected acetylated lysine residues on TLR4. The present study lays a foundation for further research on the role of lysine acetylation on TLR4 signalling. Moreover, further research is required to characterise LPCAT2 as a protein acetyltransferase.

## Introduction

Inflammation is central to many diseases such as cancer, asthma, sepsis, and cardiovascular diseases [1] and is caused by infection from various micro-organisms or by cell damage [2]. TLR4 plays a significant role in mediating inflammatory signals during bacterial infections after recognising bacterial lipopolysaccharide bound to co-receptor CD14 [3,4]. There is scientifically established information on TLR4 mechanisms of signalling and protein–protein interactions. However, recently our research group found that TLR4 co-localised with LPCAT2; this led to a change in its subcellular localisation [5]. Nonetheless, how LPCAT2 affects the subcellular localisation of TLR4 is not known. This recent finding highlights the need for further research on the molecular mechanisms of TLR4 and LPCAT2.

LPCAT2 is a lipid acyltransferase and acetyltransferase [6]. LPCAT2 is expressed significantly in peritoneal macrophages, microglia, and neutrophils [7]. Furthermore, LPCAT2 is expressed significantly in experimental allergic encephalomyelitis [8,9] and peripheral nerve injury [10–12]. Some publications suggest that LPCAT2 is a biomarker for sepsis and allergic asthma [13–15]. On the other hand, experimental conditions where LPCAT2 is silenced or inhibited result in the resolution of inflammation via decreased production of cytokines and LPCAT2 metabolites [5,10,16]. During inflammatory conditions, LPCAT2 and TLR4 expression increases in the lipid raft domain of the RAW264.7 macrophage cells, which serves as a platform for mediating inflammatory signals [5]. The enzymatic activity of LPCAT2-acylation and acetylation can influence subcellular localisation, and function is carried out on proteins [17,18]. Although acetylation commonly occurs on histones, several scientific publications have identified it on non-histone proteins [19]. Lysine acetylation can regulate protein function, interaction, and localisation [20,21]. LPCAT1, which has a very similar structure and function to LPCAT2, is known to palmitoylate histone 4, a protein [22]. Based on the finding that LPCAT1 carries out its enzymatic activity on proteins, one can hypothesize that LPCAT2 could acylate or acetylate proteins, the target being TLR4.

Since several scientific publications suggest that LPCAT2 participates in inflammation, understanding the molecular mechanisms of LPCAT2 will contribute new knowledge on inflammation and could lead to new therapies for inflammatory disorders by identifying new drug targets or biomarkers.

The present study uses computational analysis, RNA interference technology, and biochemical analysis to analyse the influence of LPCAT2 on lysine acetylation in LPS-treated RAW264.7 cells.

## Materials and methods

Chemical reagents for preparing buffers and the BCA Assay Kit were purchased from Sigma Aldrich, U.K. and Fisher Scientific, U.K. Buffers used include RIPA buffer, phosphate-buffered saline (PBS), Tris-buffered saline (TBS), blocking buffer, cell lysis buffer, elution buffer, SDS sample buffer, and ECL detection reagent [23]. PolyPlus INTERFERin was purchased from Source Bioscience, U.K. Pre-designed siRNA, Opti-MEM, Power SYBR Green, RNA to cDNA kit, and gel casting materials were purchased from Life Technologies, U.K. Antibodies, Protein A/G agarose gel beads, and protein ladders were obtained from Santa Cruz Biotechnology, UK and Cell Signalling Technologies, U.K. Recombinant mouse proteins, ELISA antibodies and detection reagents were purchased from Peprotech Ltd. DMEM culture medium and other cell culture materials were purchased from Lonza, U.K. PCR primers were designed with Primer3 Plus Bioinformatics Software and NCBI BLAST and purchased from Eurofins Genomics.

### Cell line and culture

RAW264.7 cell line was obtained from the European Collection of Cell Cultures (ECACC) through Public Health England, U.K. RAW264.7 macrophages were maintained in Dulbecco’s Modified Eagle Medium (DMEM) (Lonza, BE12-914F) supplemented with 10%(v/v) foetal bovine serum (FBS) (Labtech.com, BS-110) and 1% (v/v) 0.2 M L-glutamine (BE17-605E), and incubated at 37°C, 5% CO_2_.

### Preparation of lipopolysaccharide

Lipopolysaccharide (*Escherichia coli* O111:B4) (Sigma-Aldrich, L2630) was resuspended in LAL reagent water (<0.005 EU/ml endotoxin levels) (Lonza, W50-640). Before treating the cells with lipopolysaccharide, the cell culture medium was used to prepare the needed concentration.

### Transfection of RAW264.7 cells with LPCAT2 siRNA

RAW264.7 cells were cultured 24 h before gene silencing. Then using Opti-MEM (Reduced Serum Medium) as a diluent, a transfection mixture containing 7 nM of siRNA was prepared and added to cells. Finally, the cells were incubated at 37°C, 5% CO_2_ with Opti-MEM for 24 h for efficient gene silencing.

### Reverse transcription and real-time quantitative PCR

The reaction master mix contained 37% (v/v) nuclease-free, 230 nM of target primers (a mixture of both forward and reverse primers), 60% (v/v) Power SYBR Green, and 3 μl of ≥ 100 ng/μl cDNA. The reaction was initiated at 95°C for 10 min, then up to 40 repeated cycles of denaturing (15 s, 95°C), annealing, and extension (60 s, 60°C). GAPDH and ATP5B were used as endogenous reference genes, and relative quantification of mRNA expression was carried out using the 2^−ΔΔCt^ method [33].

### Sandwich ELISA for quantifying secreted IP10 protein

The medium from RAW264.7 cells stimulated with lipopolysaccharide was analysed for the quantity of secreted IP10 protein after 24 h. ELISA was carried out according to manufacturer’s instructions.

### Immunoprecipitation

Equal amounts of whole-cell lysates were pre-cleared and incubated with target antibodies overnight at 4°C. Then, protein A/G agarose gel beads were rinsed with PBS, added to the lysate-antibody mixture, and incubated overnight at 4°C. First, the mixture was centrifuged to separate the supernatant from protein A/G agarose beads. Next, the beads were rinsed with PBS, and then eluates were made with mild-to-harsh elution buffers [23].

### Western blot analysis

Equal amounts of whole-cell lysates and eluates were separated on pre-cast SDS-PAGE gels and blotted on to a PVDF membrane using a blot module. The blots were blocked with 0.1% bovine serum albumin in PBS-0.1% Tween 20, probed with primary and HRP-conjugated secondary antibodies. The target proteins separated on the PVDF membrane were detected and analysed using ImageJ.

### Computational biology analysis

Computational analysis of mouse protein sequences obtained from the Uniprot database was carried out using GPS-PAIL 2.0 [24] and R Programming Software (packages used were: Peptides, Biostrings, phangorn, tidyverse, ape, seqinr, rentrez, msa, and ASEB). Both gene (rentrez) and protein sequences were aligned using multiple sequence alignment (msa). The physicochemical properties of peptides were analysed using the peptides package. Phylogenetic trees were made using maximum parsimony with SeaView version 4 [25] and the phangorn package. Sequence IDs: LPCAT2- Q8BYI6, NM173014; LPCAT1- Q3TFD2, NM145376; KAT2A- Q9JHD2, NM020004; KAT2B- Q9JHD1, NM020005; CREBBP- NM001025432; ELP3- NM001253812; EP300-NM177821; KAT5- NM001362372; KAT6A- NM001081149; KAT6B- NM017479; KAT8- NM026370.

### Data and statistical analysis

Statistical analysis was carried out in R Statistical Programming Software, and graphs were plotted using the ggplot2 package. Independent experiments were repeated at least three times. Data represent mean ± standard error of mean unless stated otherwise. Paired *T*-test with Dunnett’s T3 multiple comparison tests was used for statistical analysis. All statistical tests were significant at a 95% confidence interval, *P*≤0.05.

## Results

### Transfection of RAW264.7 cells with LPCAT2 siRNA does not affect the basal function of RAW264.7 cells

**Figure 1:**
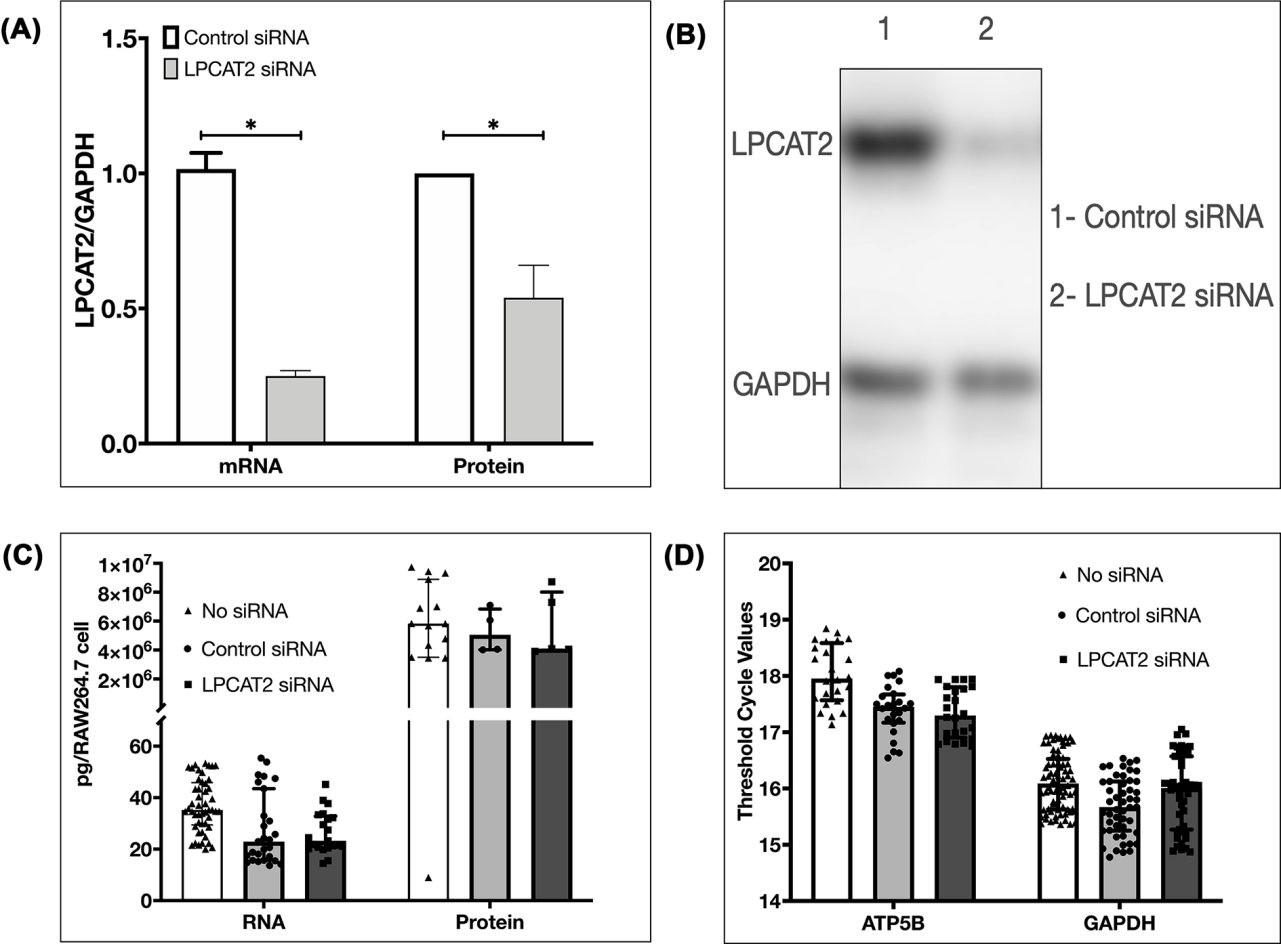
Effect of LPCAT2 knockdown on RAW264.7 cells Transfection of RAW264.7 cells with LPCAT2 siRNA causes ≥70% decrease in LPCAT2 gene expression and ≥50% decrease in LPCAT2 protein expression (**A**) *P≤0.05. Fold change shows the optical density of LPCAT2 normalised to GAPDH. Data represent mean of at least three independent experiments (*n*≥3) ± standard error (A) of Western blots (**B**) Transfection of siRNA into RAW264.7 cells does not cause a significant difference in total RNA and total protein (**C**), and in the expression of housekeeping genes ATP5B and GAPDH (**D**) when compared with cells with no transfected siRNA. Data represent the median of at least three independent experiments (*n*≥3) ± interquartile range.

The gene and protein expression of LPCAT2 were analysed to confirm the knockdown of LPCAT2. Figure 1A shows that the LPCAT2 gene is significantly lower in cells transfected with LPCAT2 siRNA (0.25 ± 0.02, *P*=0.0015). Likewise, LPCAT2 protein decreased in cells transfected with LPCAT2 siRNA (0.54 ± 0.12, *P*=0.03).

Analysis of the total amount of RNA and protein and the gene expression of housekeeping genes-ATP5B and GAPDH determined the basal function of RAW264.7 cells after transfecting the cells with LPCAT2 siRNA. Figure 1C shows that transfection of RAW264.7 cells with LPCAT2 siRNA does not significantly affect the total amount of RNA (23.25 pg/RAW264.7 cell; 20.56 pg/RAW264.7 cell to 32.78 pg/RAW264.7 cell) when compared with non-transfected cells (35.2 pg/RAW264.7 cell; 29.39 pg/RAW264.7 cell to 45.84 pg/RAW264.7 cell, *P*=0.98) and the total amount of protein (4.09 × 10^6^ pg/RAW264.7 cell; 3.98 × 10^6^ pg/RAW264.7 cell to 8.01 × 10^6^ pg/RAW264.7 cell) when compared with non-transfected cells (5.82 × 10^6^ pg/RAW264.7 cell; 3.5 × 10^6^ pg/RAW264.7 cell to 8.89 × 10^6^ pg/RAW264.7 cell, *P*=0.98). Likewise, Figure 1D shows that transfection of RAW264.7 cells with LPCAT2 siRNA does not significantly affect the gene expression of housekeeping gene ATP5B (17.3; 16.9–17.8) when compared with non-transfected cells (17.96; 17.57–18.58, *P*=0.95) and GAPDH (16.01; 15.27–16.57) when compared with non-transfected cells (16.09; 16.52–15.63, *P*=0.95).

### Analysis of the relatedness of LPCAT2 to commonly known lysine acetyltransferases (KATs)

**Figure 2:**
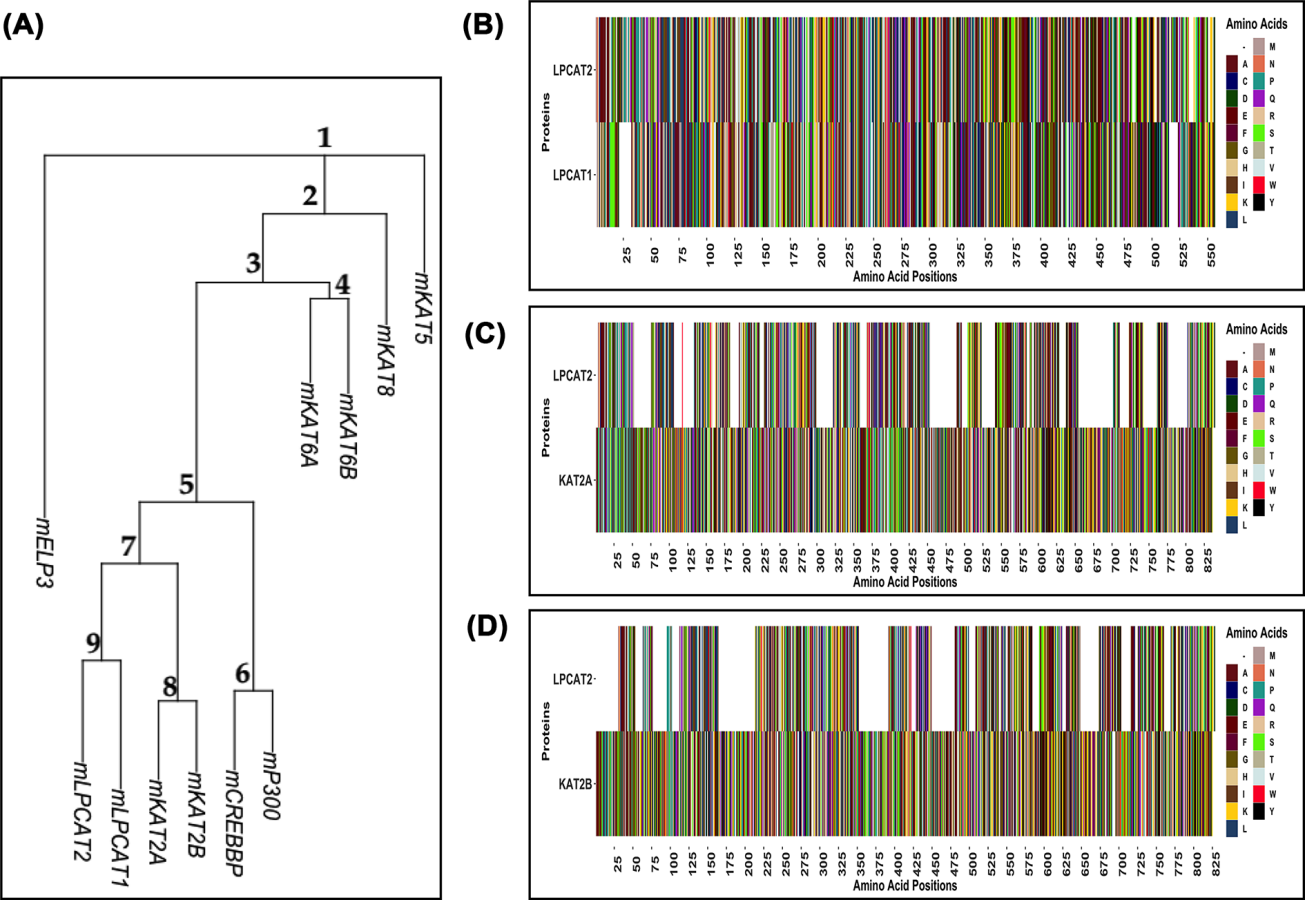
Analysis of the relatedness of LPCAT2 To other lysine acetyltransferases in mice (**A**) Phylogenetic tree showing the degree of relatedness of LPCAT1 gene, LPCAT2 gene, and genes of other KATs. Numbers indicate node positions. (**B–D**) Sequence alignment of proteins in node 7, white space indicates gaps in alignment. Letters symbolising amino acids are IUPAC standards.

Using R Statistical Programming Software, the genetic relatedness of LPCAT2 to other KATs was analysed. LPCAT1 is a positive control because it is a very similar protein to LPCAT2. Indeed, Figure 2A shows that LPCAT2 and LPCAT1 belong to the same family. Moreover, it suggests that LPCAT2 and LPCAT1 belong to the same superfamily as KAT2A and KAT2B. Therefore, the similarity of the protein sequences was analysed by aligning LPCAT2 to each protein from node 7 in Figure 2A. Figure 2B–D shows that LPCAT2 versus LPCAT1 has less than 5% gaps in alignment (B), whereas LPCAT2 versus KAT2A (C) and LPCAT2 versus KAT2B (D) have less than or equal to 35% gaps in the alignment.

### LPCAT2 influences pan-lysine acetylation in RAW264.7 cells

**Figure 3:**
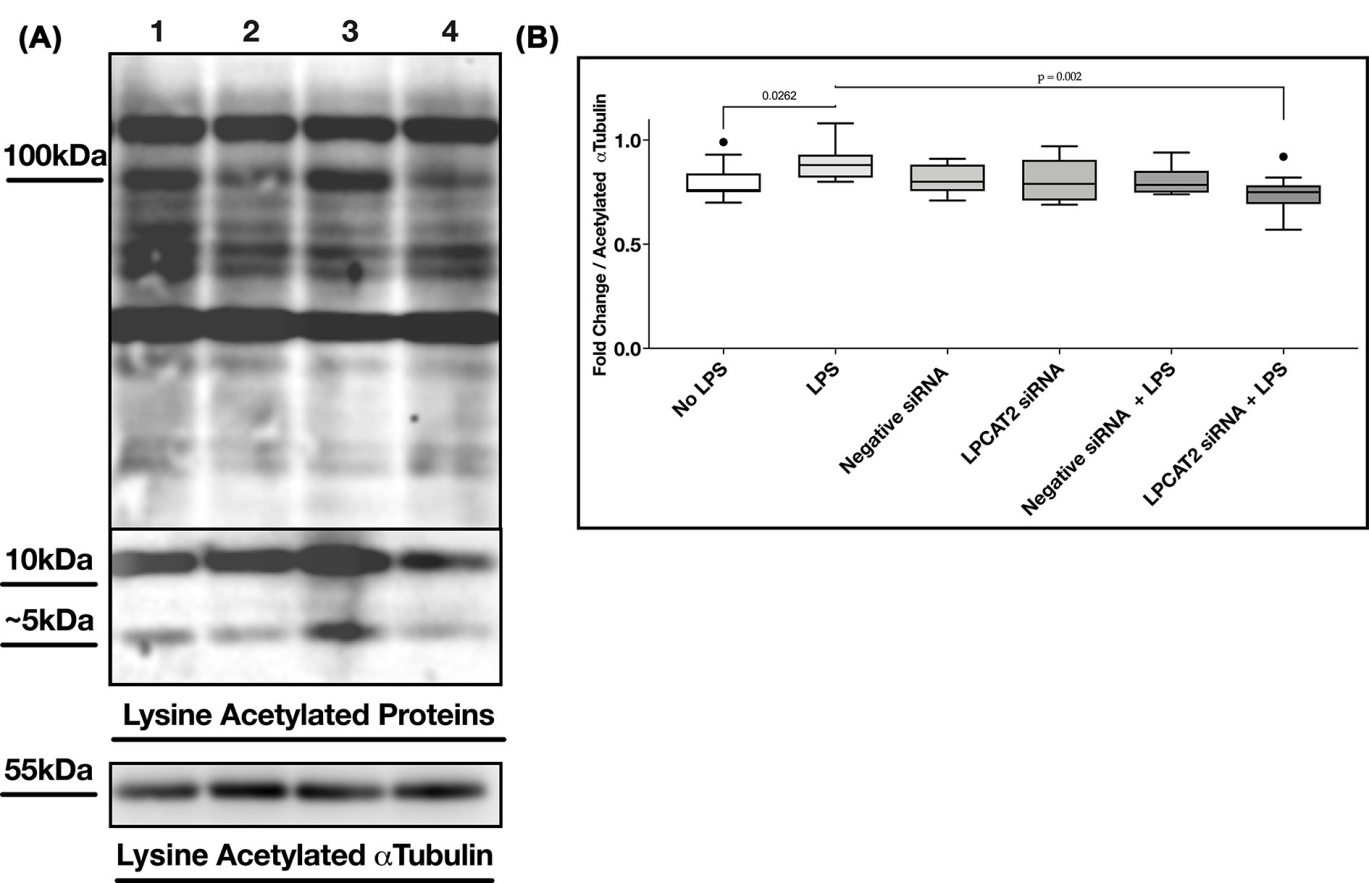
Analysis of pan-lysine acetylation in lipopolysaccharide-stimulated RAW264.7 cells (**A**) Western blot image of lysine acetylated proteins. Acetylated α-tubulin was used as a positive control. Lane 1: Control siRNA, Lane 2: LPCAT2 siRNA, Lane 3: Control siRNA + LPS, Lane 4: LPCAT2 siRNA + LPS. (**B**) Fold change in optical density of lysine acetylated proteins normalised to acetylated α-tubulin. Mid-bars represent median, boxes represent interquartile range, upper bar represent maximum, and lower bar represent minimum. Welch’s *T*-test *P* values; No LPS versus LPS (*P*=0.03), No LPS versus negative siRNA (*P*=0.73), LPS versus LPCAT2 siRNA + LPS (*P*=0.002). Data were obtained from at least three independent experiments.

Acetylated lysine residues were detected using acetylated lysine antibodies. Figure 3 shows that stimulating RAW264.7 cells with LPS increased the density of some protein bands with acetylated lysine; this is visible around 10 kDa. The box plots show fold change after normalising optical densities to acetylated α-tubulin densities. Some protein bands (100 and ∼5 kDa) showed reduction after the knockdown of LPCAT2 with or without LPS (Figure 3A). The increase in lysine acetylation after LPS treatment is statistically significant (*P*=0.03). Likewise, the decrease in lysine acetylation after LPCAT2 knockdown (*P*=0.002). This result indicates that LPCAT2 silencing results in lower band intensities of lysine-acetylated proteins.

### *In silico* prediction of lysine acetylation of lipopolysaccharide receptors

Due to the presence of LPS-inducible acetylated lysine at 100 kDa, the protein sequences of mouse TLR4, MD2, and CD14 were analysed for the possible presence of lysine residues. Two software were used for this analysis: GPS-PAIL version 2.0 and ASEB. Table 1 shows GPS-PAIL version 2.0 software predicted that TLR4 is acetylated on the lysine residue at position 817 by CREBBP, but CD14 and MD2 did not show any possibility for lysine acetylation. Therefore, RelA was used as a positive control, as it is already experimentally proven to undergo lysine acetylation [26].

**Table 1:** *In silico* prediction of lysine acetylation in lipopolysaccharide receptors using GPS-PAIL version 2.0

Protein	Position	Peptide	Score	Cut-off	Acetyltransferase
CD14	NA	NA	NA	NA	NA
MD2	NA	NA	NA	NA	NA
TLR4	817	KNALLDGKASNPEQ	2.09	1.79	CREBBP
RelA	310	KRTYETFKSIMKKS	2.32	1.79	CREBBP
RelA	122	NLGIQCVKKRDLEQ	2.08	1.69	KAT2B

NA indicates that no sites of lysine acetylation was detected. Position indicates the position of the lysine with predicted acetylation. The acetyltransferases were also predicted based on known information about binding sites of these enzymes. The score indicates the probability of acetylation. The higher the score, the higher the chance of acetylation. The cut-off is the number under the threshold. This analysis was carried out using a high threshold. Red letters signify the supposed position of lysine acetylation.

Further analysis of mouse TLR4 protein sequence using ASEB revealed the possibility of more than one lysine residue undergoing lysine acetylation. In Table 2, the lysine residue at position 817 was predicted to undergo lysine acetylation by CREBBP and lysine residues at positions 367 and 503. The lysine residue at position 152 was predicted to undergo lysine acetylation by KAT2A and or KAT2B.

**Table 2:** *In silico* prediction of lysine acetylation of TLR4 using ASEB

Position	Peptide	Acetyltransferase
152	PIGQLITLKKLNVAHNF	KAT2A, KAT2B
367	NKGSISFKKVALPSLSY	CREBBP
503	NLTFLDLSKCQLEQISW	CREBBP
817	KNALLDGKASNPEQ	CREBBP

ASEB predicted more peptides to undergo lysine acetylation in TLR4. However, the peptides selected and shown in this table all have a *P*-value of* P*≤0.1.

Each peptide predicted to contain acetylated lysine residue was analysed to understand their properties and predict their potential roles. As shown in Table 3, peptides were globular, suggesting that the peptides are situated either on the extracellular or the intracellular end of TLR4 but not in the transmembrane region. Furthermore, the high aliphatic index values also predict that these peptides are thermostable.

**Table 3:** Properties of predicted lysine acetylated peptides in TLR4

Position	Peptide	HI	AI	Membrane position
152	PIGQLITLKKLNVAHNF	0.29	137.7	Globular
367	NKGSISFKKVALPSLSY	-0.05	91.8	Globular
503	NLTFLDLSKCQLEQISW	0.02	114.7	Globular
817	KNALLDGKASNPEQ	-1.17	65.3	Globular

Hydrophobicity index (HI) indicates a lipophilic or hydrophilic peptide. The aliphatic index (AI) indicates thermostability. All peptides are predicted to be globular because of their low hydrophobicity index.

### Acetylated lysine detected on TLR4 by immunoprecipitation and Western blot

**Figure 4:**
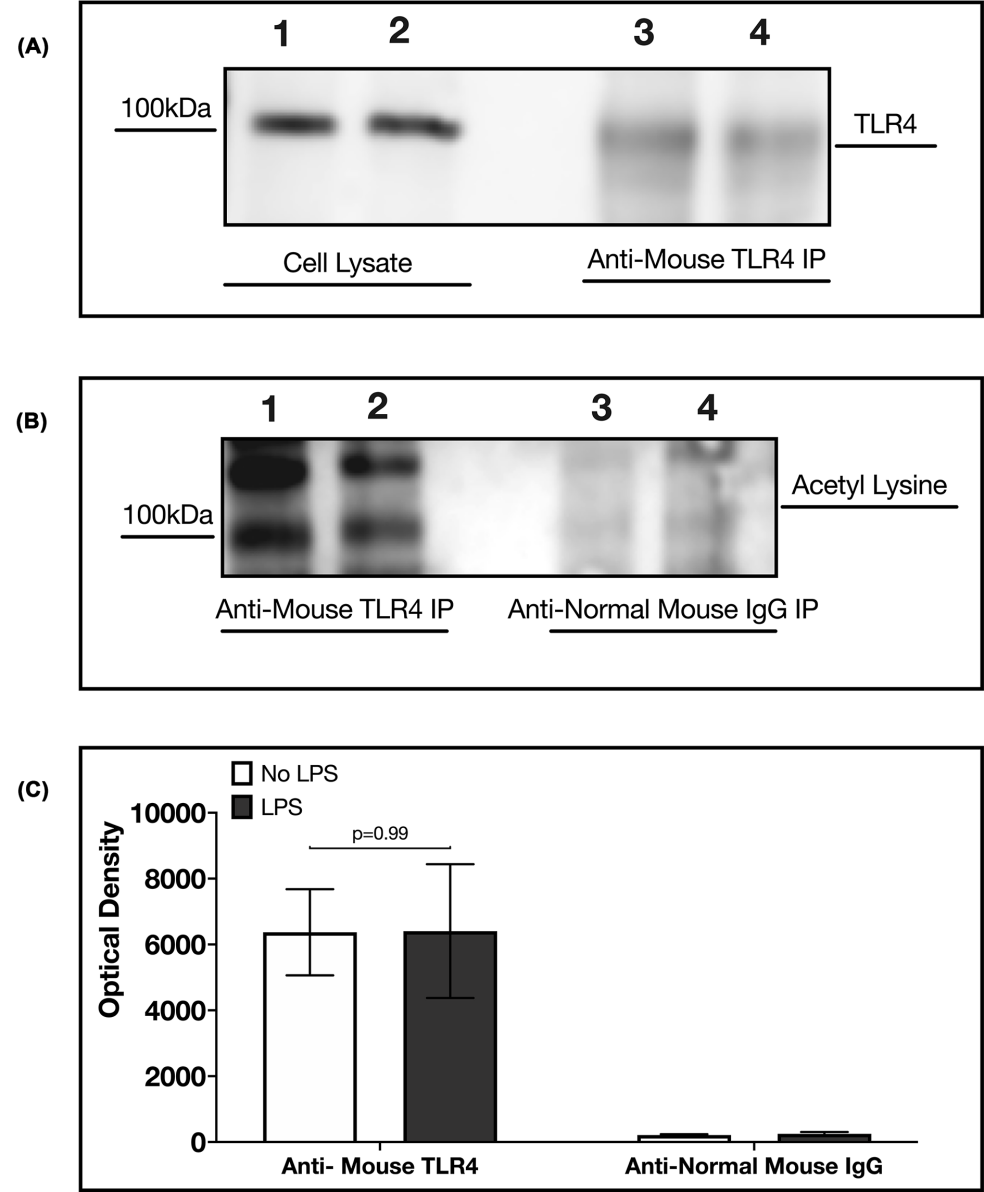
Detection of acetylated lysine on TLR4 in RAW264.7 cells (**A**) Western blot of TLR4 in after immunoprecipitation of TLR4. Lane 1: Cleared cell lysate (No LPS treatment); Lane 2: Cleared cell lysate (LPS treatment); Lane 3: TLR4 eluate (no LPS treatment); Lane 4: TLR4 eluate (LPS treatment). (**B**) Western blot of acetylated lysine after immunoprecipitation of TLR4. Lane 1: TLR4 eluate (no LPS treatment); Lane 2: TLR4 eluate (LPS treatment); Lane 3: normal mouse IgG eluate (no LPS treatment); Lane 4: normal mouse IgG eluate (LPS treatment); Normal mouse IgG is an isotype control for immunoprecipitation. (**C**) Optical densities of acetylated lysine detected on TLR4. The optical densities were normalised to total TLR4 (optical density of TLR4 in cleared cell lysate + optical density of TLR4 in TLR4 Eluate). Welch’s *T*-test shows no significant difference between no LPS treatment and LPS treatment (*P*=0.99). Data are obtained from three independent experiments.

Further analysis to confirm the presence of lysine acetylation in TLR4 was carried out by immunoprecipitating TLR4 and blotting for acetylated lysine residues. Figure 4A,B show that at 100 kDa, TLR4 was detected in both blots using TLR4 antibodies and acetylated lysine antibodies after immunoprecipitation of TLR4. However, in Figure 4C, the treatment of RAW264.7 cells with LPS did not show any significant difference (*P*=0.99).

To ensure that the detected protein is TLR4, we silenced the expression of TLR4 in RAW246.7 cells and repeated the analysis. In this case, acetylated lysine proteins were immunoprecipitated, and eluates were analysed for the presence of TLR4. The postulation was that if the detected protein were not TLR4, it would not decrease when TLR4 is silenced, and the TLR4 antibody would not detect it. In Supplementary Figure S4c, we see again that TLR4 was detected from the eluate of lysine-acetylated proteins by TLR4 antibody. Supplementary Figure S4c shows that knockdown of TLR4 reduced the presence of lysine acetylation on TLR4 by ≥50%. This result confirms that the protein detected in TLR4 strengthens evidence of lysine acetylation on TLR4. TLR4 expression was also reduced in cells not treated with LPS but transfected with TLR4 siRNA [23].

### LPCAT2 regulates the expression of interferon-β and interferon-inducible protein 10

**Figure 5:**
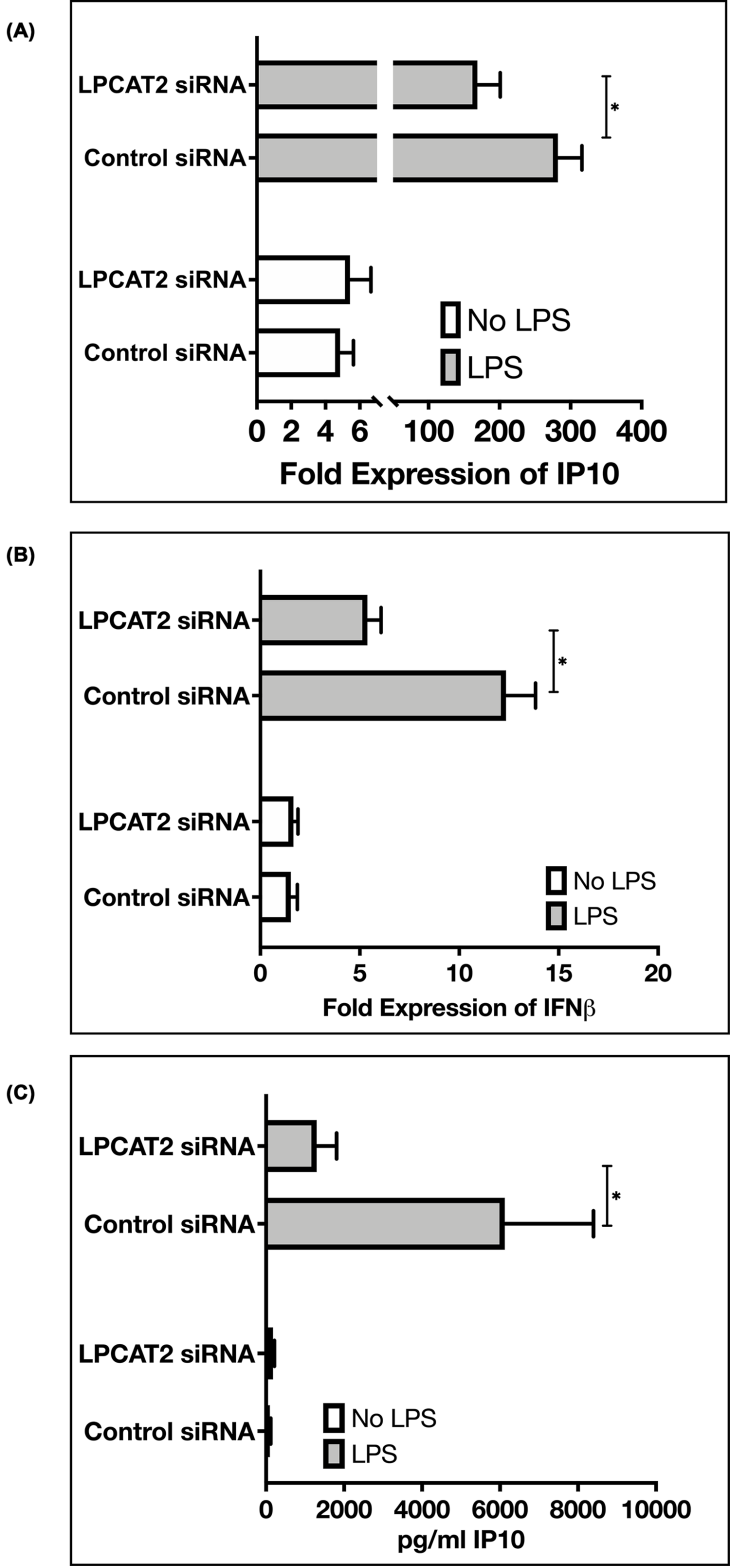
Effect of LPCAT2 knockdown on mRNA expression IFNβ and IP10 Knockdown of LPCAT2 affects the gene expression of IFNβ (**A**) and interferon-inducible protein 10, IP10 (**B**) and protein expression of IP10 (**C**) in RAW264.7 cells stimulated with TLR4 ligand (100 ng/ml of *E. coli* O111:B4 lipopolysaccharide). Non-treated RAW264.7 cells did not show any significant difference. Welch’s *T*-test (*P≤0.05). Data represent the mean of at least three independent experiments (*n*≥3) ± standard error.

A recent publication has shown that LPCAT2 can regulate the expression of pro-inflammatory cytokines; TNFα and IL6 [5]; however, interferon-β (IFNβ) and interferon-inducible protein 10 (IP10), which are dependent on TLR4-Trif signalling pathway, were not analysed. Figure 5B shows that knockdown of LPCAT2 results in a significant decrease (5.38 ± 0.69, *P*=0.00025) in the gene expression of IFNβ in cells stimulated with 100 ng/ml lipopolysaccharide. IP10, which can also be induced by IFNβ, was analysed. Figure 5A,C shows that knockdown of LPCAT2 significantly decreased the gene (168.7 ± 32.27, *P*=0.021) and protein (1299.84 ± 507.87, *P*=0.04) expression of IP10. In non-treated cells, knockdown of LPCAT2 did not effect a significant change in IFNβ gene (*P*=0.46), IP10 gene (*P*=0.97), and IP10 protein (*P*=0.10) expression.

### Preliminary result showing LPCAT2 reduces lysine acetylation detected on TLR4

**Figure 6:**
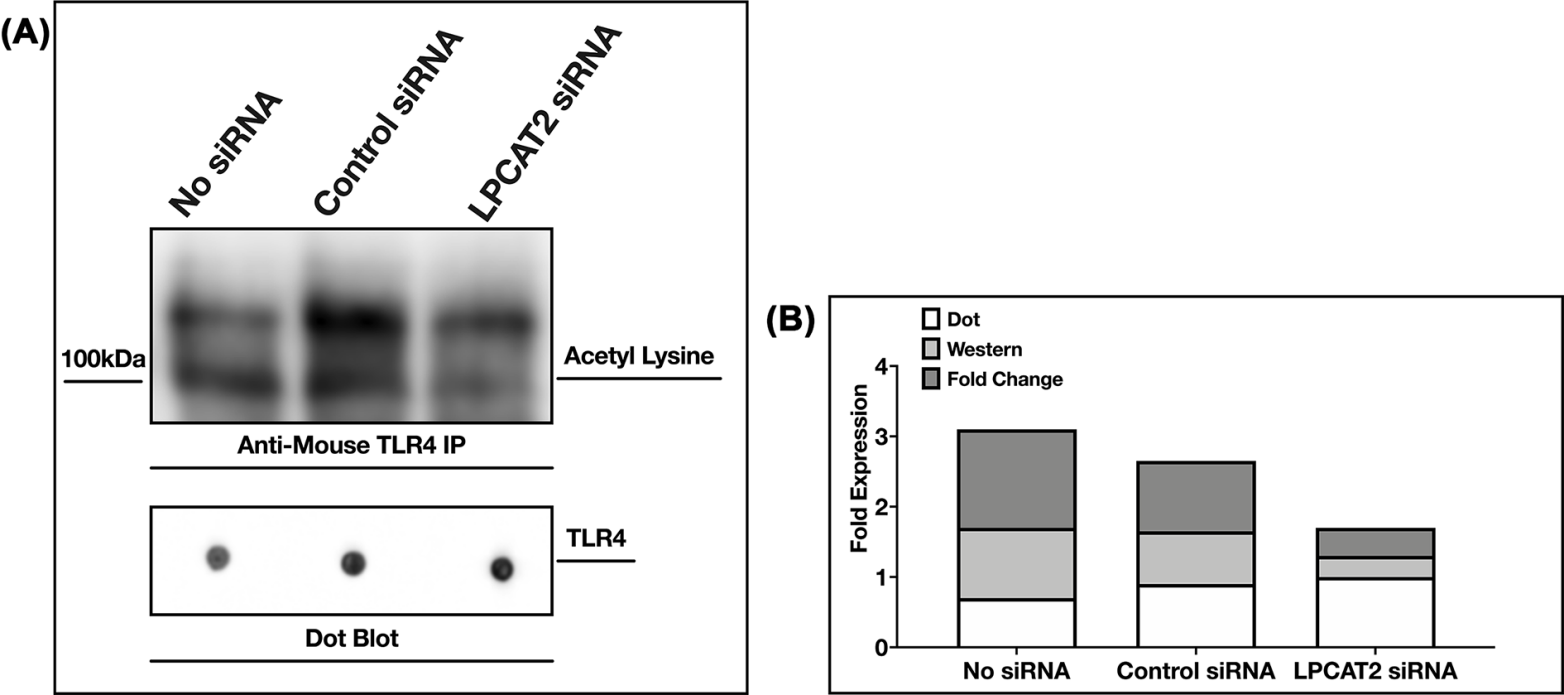
Knockdown of LPCAT2 reduces detected acetylated lysine residues on TLR4 (**A**) Acetylated lysine blots (WB) of TLR4 precipitates (IP). Lane 1: No siRNA, Lane 2: Control siRNA, Lane 3: LPCAT2 siRNA. TLR4 dot blots of same quantity of proteins from TLR4 eluates were used as controls of protein amounts. (**B**) Optical densities of blots in (A). Data are obtained from one experiment.

As the initial study was to understand the role of LPCAT2 on lysine acetylation, we sought to determine the influence of LPCAT2 on the detected lysine acetylation on TLR4. Figure 6 shows a TLR4 blot of acetylated lysine eluates from RAW264.7 cells. The optical densities in Figure 6 shows *a* ≥ 50% decrease in acetylated TLR4 lysine after the knockdown of LPCAT2. However, as these data are obtained from one experiment, further studies will be required to establish this result.

## Discussion

LPCAT2 is commonly known as a lipid-modifying enzyme. However, there is scientific evidence that LPCAT1, which is similar in structure and function to LPCAT2, palmitoylates histone 4 [22]. Therefore, LPCAT2 may modify proteins. Moreover, LPCAT2 co-localises with TLR4 and modifies its subcellular localisation and function through an unknown mechanism [5]. Adding acyl groups to proteins can regulate their subcellular localisation and function [27]. Our results suggest that TLR4-LPCAT2 co-localising may result in the lysine acetylation of TLR4, which may influence its subcellular localisation. Moreover, our results show that LPCAT2 influences lysine acetylation in LPS-treated RAW264.7 cells.

### LPCAT2 gene and protein sequence is homologous to other lysine acetyltransferases

About 50–70% homology is required to conserve enzyme functions [28]. Figure 2 shows that LPCAT2 shares at least 65% similarity with KAT2A and KAT2B, established lysine acetyltransferases. Moreover, analysis of their gene sequences showed that LPCAT2, LPCAT1, KAT2A, and KAT2B evolved from the same superfamily. The results from this computational analysis lay a foundation for further scientific experiments required to classify LPCAT2 as a lysine acetyltransferase.

### LPCAT2 influences pan-acetylated lysines after LPS treatment in RAW264.7 cells

#### The housekeeping genes

ATP5B and GAPDH and the total RNA and protein amount did not change when RAW264.7 cells were transfected with siRNA (Figure 1). This result eliminates the possibility that the observed gene expression or protein expression results from overall changes in the cell.

Palmitoylation can regulate the subcellular localisation and signalling of TLR2. It is also present on other TLRs like TLR5 and TLR10 but not TLR4 [29]. As TLR4 was not found amongst palmitoylated proteins, we explored the next possibility, which is acetylation. LPCAT2 has both acyltransferase and acetyltransferase [6]. However, unlike LPCAT1, no previously published evidence suggests that LPCAT2 can carry out its enzymatic activities on proteins.

In Figure 3, we find a significant increase in pan lysine acetylation after treating RAW264.7 cells with LPS. Similarly, a study of global lysine acetylome in HepG2 cells showed increased lysine acetylation after LPS treatment [32]. Figure 3 also shows for the first time that when LPCAT2 was knocked down in RAW264.7 cells and the cells were treated with LPS, the optical densities of lysine-acetylated proteins decreased significantly.

### Lysine acetylation detected on TLR4

There is a visible decrease in the optical density of bands at 100 kDa when LPCAT2 is silenced (Figure 3A). Except for TLR4, many other proteins have an approximate mass of 100 kDa. On the UniProt database [30], there are about 3500 proteins between 90 and 110 kDa. Only about 25 of these proteins have published evidence of lipidation. Therefore, a more TLR4-specific study was required to confirm its lysine acetylation. Computational analysis to predict TLR4 lysine acetylation suggests that TLR4 may undergo lysine acetylation on lysines 152, 367, 503, and 817 catalysed by KAT2A, KAT2B, or CREBBP (Tables 1 and 2).

Furthermore, biochemical analysis shows the presence of lysine acetylation on TLR4 (Figure 4). However, further experiments will be required to know the specific lysine residues acetylated on TLR4 and their role in regulating TLR4 function. In Table 3, we predicted that the TLR4 peptides with acetylated lysine residues are hydrophilic and globular. Although these are *in silico* predictions, the results suggest that lysine acetylation may be occurring in the extracellular domain of TLR4, where it binds to its ligands or the intracellular domain of TLR4, where it binds to other proteins and transmits signals. Indeed, a thesis suggested that TLR4 lysine acetylation influences its interaction with its adaptor proteins MyD88, TRAM, and TRIF, which eventually affect the production of inflammatory cytokines [31]. We have previously shown that knockdown of LPCAT2 reduces MyD88-dependent cytokines after LPS–TLR4 interaction [5]. Likewise, Figure 5 shows that the knockdown of LPCAT2 influences TRIF-dependent cytokines. TLR4 protein or gene expression is not affected by silencing LPCAT2 (data for TLR4 gene expression not shown). However, Figure 6 suggests for the first time that LPCAT2 may influence lysine acetylation detected on TLR4.

In conclusion, the results from the present study show that LPCAT2 influences lysine acetylation in RAW264.7 cells treated with LPS. Moreover, the results also show that TLR4 undergoes lysine acetylation, and LPCAT2 may influence the detected lysine acetylation.

These novel results lay a foundation for further research on the role of lysine acetylation on TLR4 signalling and the characterisation of LPCAT2 as a protein acetyltransferase.

## Highlights

- LPCAT2 gene silencing influences lysine acetylation in RAW264.7 cells after treatment with LPS.
- Computational analysis predicts lysine acetylation on TLR4.
- Biochemical analysis reveals lysine acetylation on TLR4 in RAW264.7 cells.
- Computational analysis shows relatedness of LPCAT2 to lysine acetyltransferases; KAT2A and KAT2B.
- Preliminary study shows that LPCAT2 gene silencing decreases lysine acetylation found on TLR4 in RAW264.7 cells.

## Supplementary Material

Supplementary Figures S1-S6

Supplementary Data

## Data Availability

Supplementary data have been submitted with this manuscript. For more information on data availability, please contact the corresponding author at the email address provided.

## Abbreviations

AI: aliphatic index
ASEB: acetylation set enrichment-based
ATP5B: adenosine priphosphate synthase β subunit
CD14: cluster of differentiation 14
CREBBP: cyclic adenosine monophosphate response element -binding protein (CREB) binding protein
ECACC: European Collection of Cell Cultures
HI: hydrophobicity index
IFNβ: interferon-β
IP10: interferon-inducible protein 10
KAT: known lysine acetyltransferase
LAL: limulus amebocyte lysate
LPCAT2: lysophosphatidyl choline acetyltransferase 2
LPS: lipopolysaccharide
TLR4: Toll -like receptor 4
TRAM: translocating chain-associating membrane protein
TRIF: TIR-domain-containing adapter-inducing interferon-β
