# Supplementary material for: Possible regulation of Toll-like receptor 4 by lysine acetylation through LPCAT2 activity in RAW264.7 cells
Source: Biosci Rep. 2022 Jul 15;42(7):BSR20220251. doi: 10.1042/BSR20220251 (PMC9289797; doi:10.1042/BSR20220251)
Supplement: Supplementary Figures S1-S6 [file BSR-2022-0251_supp.pdf]

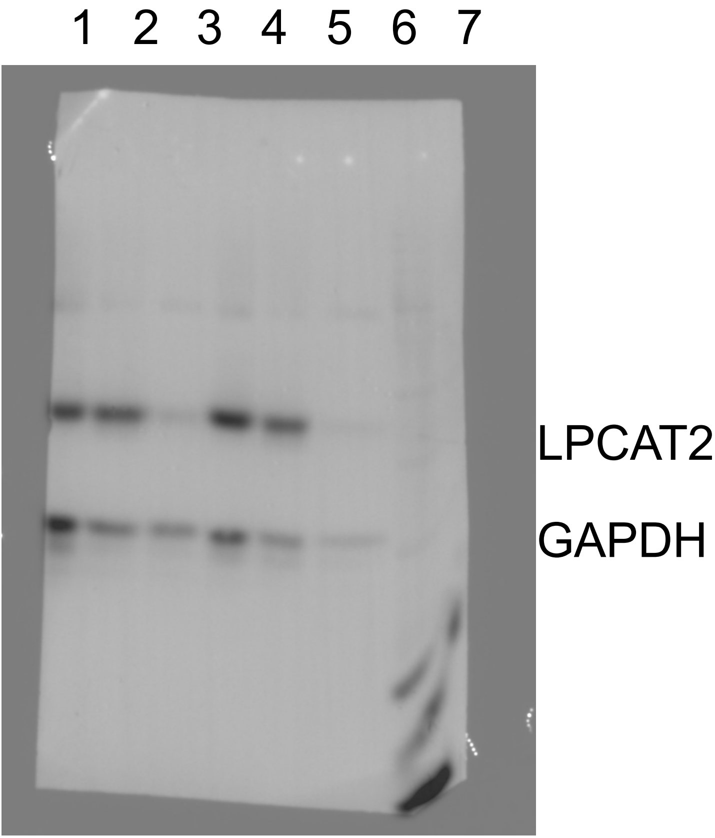

Figure S1c: Knockdown of LPCAT2 in RAW264.7 cells normalised. 1 – Untreated; 2 – Control siRNA; 3 – LPCAT2 siRNA; 4 - LPS; 5 – Control siRNA + LPS; 6 – LPCAT2 siRNA + LPS; 7 – Molecular Weight Marker. Lanes 2 and 3 have been cropped and shown in figure 1c.

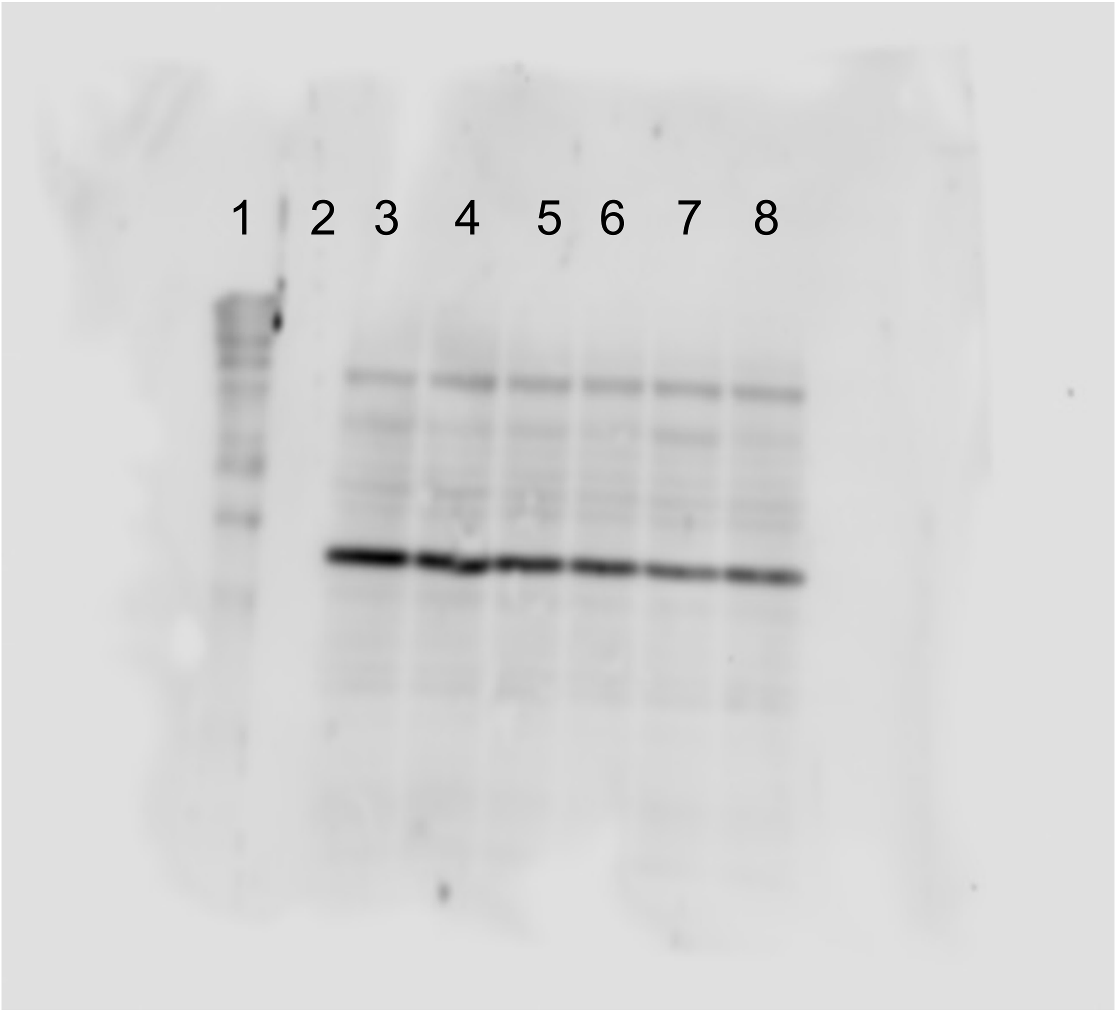

Figure S3a_1: Blot of proteins with acetylated lysine in RAW264.7 cells. 1- Molecular weight Marker; 2- Blank; 3- No treatment; 4 – LPS; 5- Control siRNA; 6- LPCAT2 siRNA; 7- Control siRNA + LPS; 8- LPCAT2 siRNA + LPS. Lanes 5 to 8 have been cropped and shown in figure 3a.

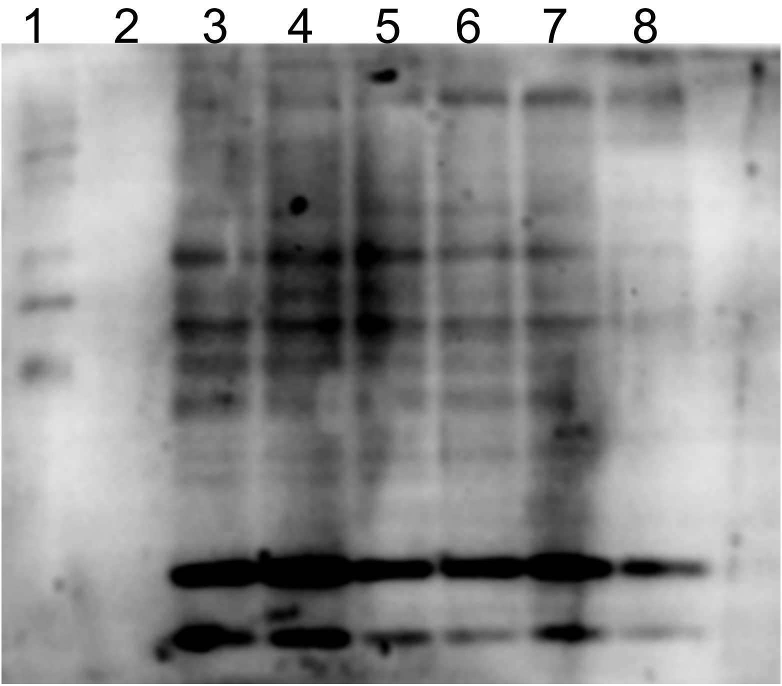

Figure S3a_2: Blot of proteins with acetylated lysine in RAW264.7 cells. 1- Molecular weight Marker; 2 – Blank; 3 – No treatment; 4 – LPS; 5 – Control siRNA; 6 – LPCAT2 siRNA; 7 – Control siRNA + LPS; 8 – LPCAT2 siRNA + LPS. Lanes 5 to 8 have been cropped and shown in figure 3a.

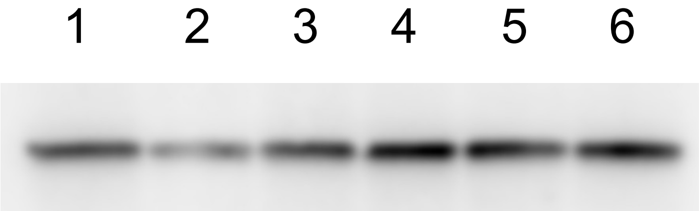

Figure S3a_3: Blot of acetylated alpha tubulin in RAW264.7 cells. 1 – No treatment; 2 – LPS; 3 – Control siRNA; 4 – LPCAT2 siRNA; 5 – Control siRNA + LPS; 6 – LPCAT2 siRNA + LPS. Lanes 3 to 6 have been cropped and shown in figure 3a.

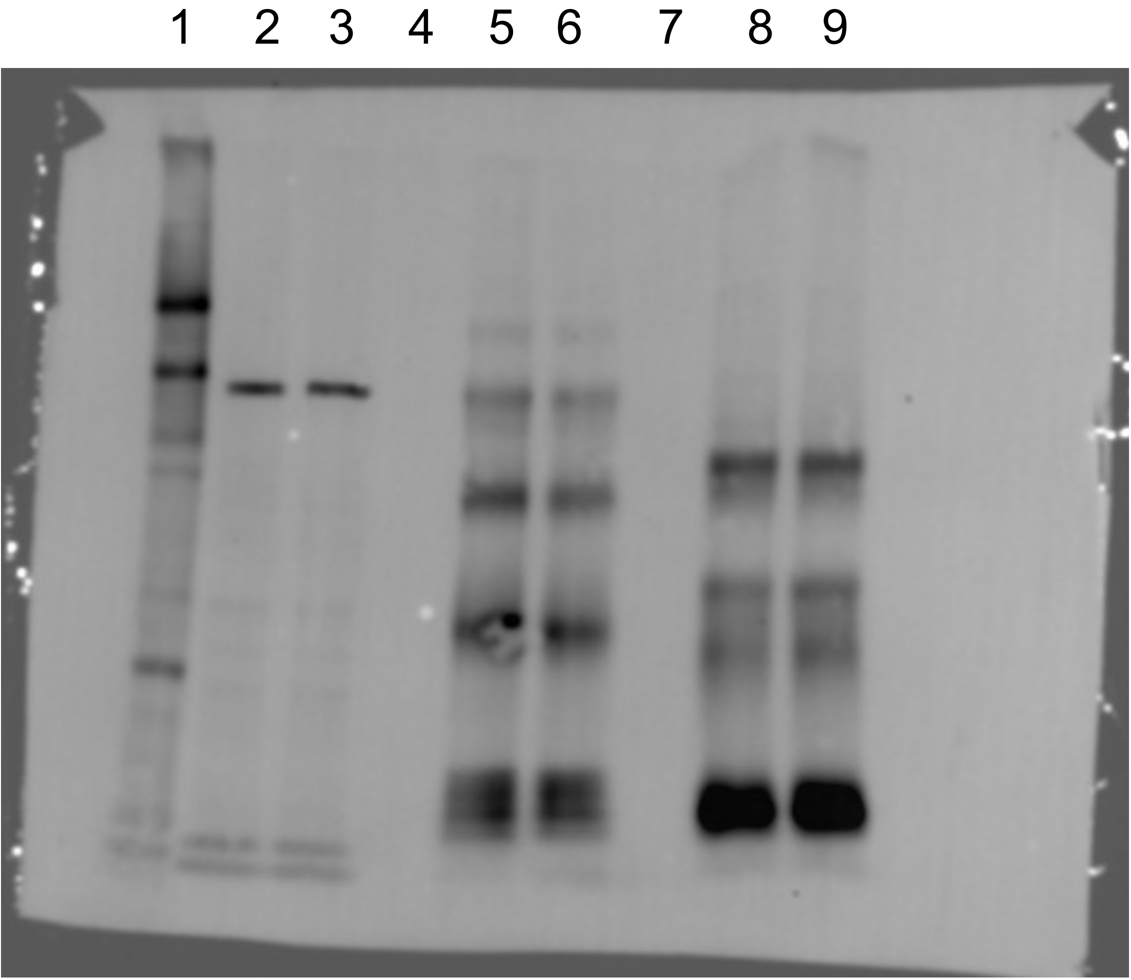

Figure S4a_1: Blot of TLR4 immunoprecipitate with or without LPCAT2 knockdown. 1 – Molecular weight marker; 2 – Control siRNA (Whole cell lysates); 3 – LPCAT2 siRNA (Whole cell lysates); 4 – Blank; 5 – Control siRNA (TLR4 eluate); 6 – LPCAT2 siRNA(TLR4 eluate); 8 – Control siRNA (IgG eluate); 9 – LPCAT2 siRNA(IgG eluate).

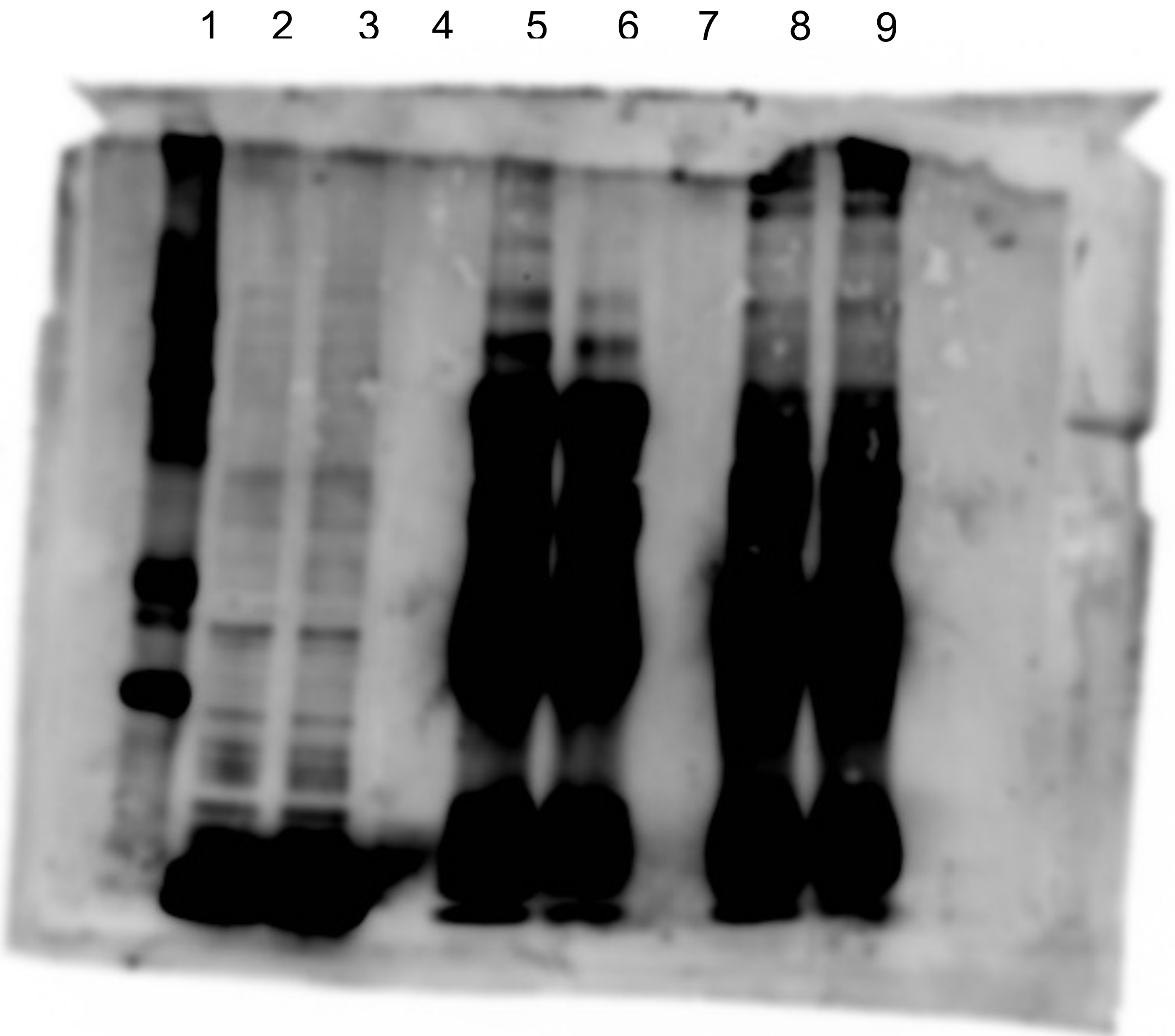

Figure S4a_2: Blot of TLR4 immunoprecipitate with or without LPCAT2 knockdown. 1 – Molecular weight marker; 2 – No treatment (cell lysate); 3 – LPS (cell lysate); 4 – Blank; 5 – Control siRNA (TLR4 eluate); 6 – LPCAT2 siRNA(TLR4 eluate); 7 – Blank; 8 – Control siRNA (IgG eluate); 9 – LPCAT2 siRNA(IgG eluate). Lanes 5 to 9 have been cropped and shown as part of figure 4a.

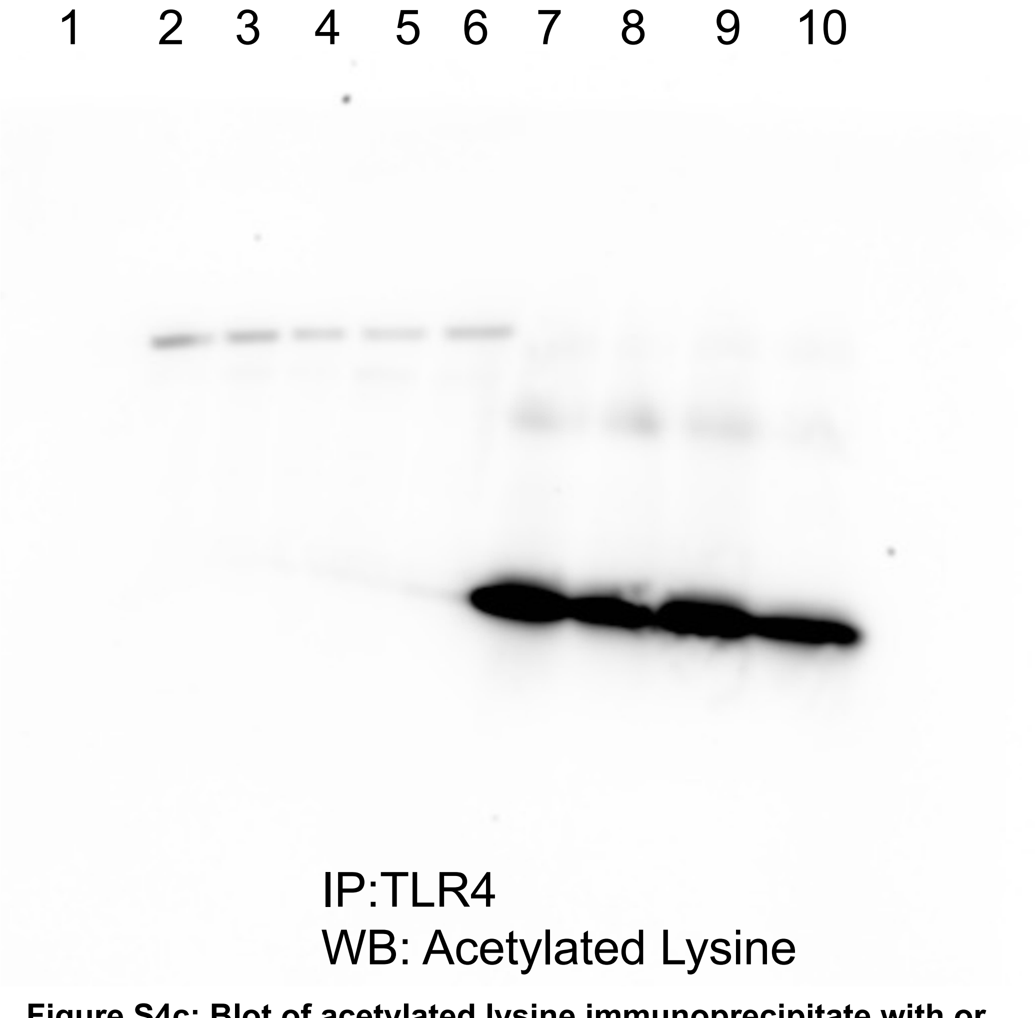

Figure S4c: Blot of acetylated lysine immunoprecipitate with or without TLR4 knockdown. 1 – Non-biotinylated Molecular weight marker; 2 – No treatment; 3 – Control siRNA; 4 – TLR4 siRNA; 5 – Control siRNA + LPS; 6 –TLR4 siRNA + LPS; 7 – Control siRNA; 8 – TLR4 siRNA; 9 – Control siRNA + LPS; 10 – TLR4 siRNA + LPS.

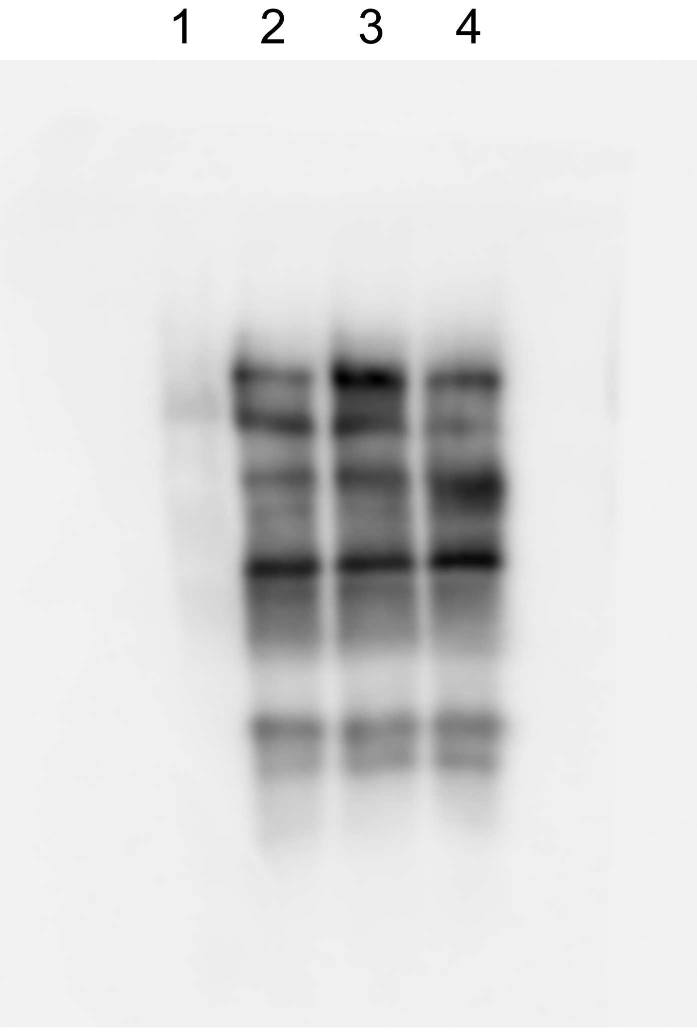

Figure S6a_1: Acetylated lysine blots of TLR4 Eluates. 1 – Molecular weight marker; 2 – No siRNA; 3 – Control siRNA; 4 – LPCAT2 siRNA.

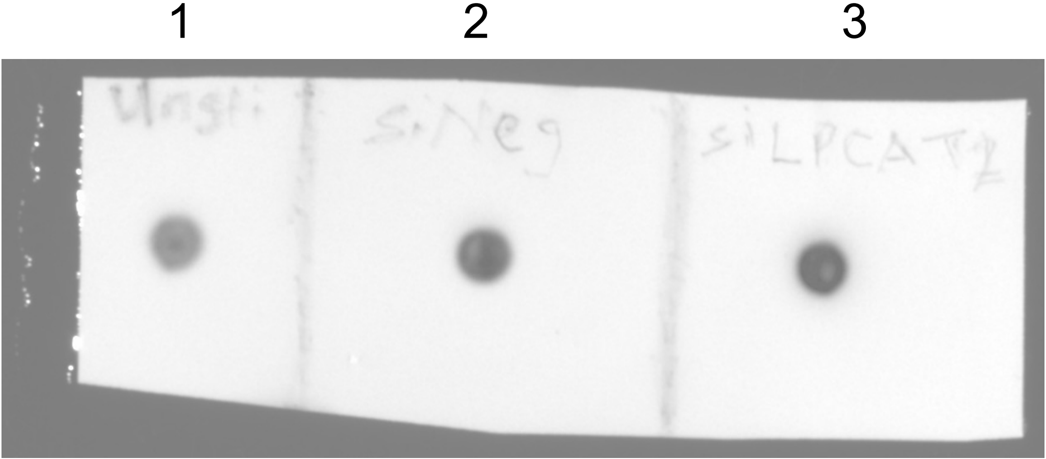

Figure S6a_2: TLR4 dot blots of TLR4 eluates. 1- No siRNA; 2- Control siRNA; 3- LPCAT2 siRNA.
